# Mucoadhesive film containing α-mangostin shows potential role in oral cancer treatment

**DOI:** 10.1186/s12903-021-01845-0

**Published:** 2021-10-10

**Authors:** Piyawat Tangsuksan, Jureeporn Chuerduangphui, Chutha Takahashi Yupanqui, Teerapol Srichana, Ekarat Hitakomate, Chamsai Pientong, Tipaya Ekalaksananan, Wipawee Nittayananta

**Affiliations:** 1 Faculty of Dentistry, Thammasat University, Pathum Thani, Thailand; 2 Department of Microbiology, Faculty of Science, Kasetsart University, Bangkok, Thailand; 3 Center of Excellence in Functional Foods and Gastronomy, Faculty of Agro-Industry, Prince of Songkla University, Hat Yai, Songkhla, Thailand; 4 Drug Delivery System Excellence Center, Faculty of Pharmaceutical Sciences, Prince of Songkla University, Hat Yai, Songkhla, Thailand; 5 Department of Pharmaceutical Technology, Faculty of Pharmaceutical Sciences, Prince of Songkla University, Hat Yai, Songkhla, Thailand; 6 Department of Microbiology, Faculty of Medicine, Khon Kaen University, Khon Kaen, Thailand; 7 HPV and EBV and Carcinogenesis Research Group, Khon Kaen University, Khon Kaen, Thailand

**Keywords:** Human papillomavirus, α-mangostin, Oral squamous cell carcinoma, Potentially malignant disorder, Wound healing

## Abstract

**Background:**

Oral cancer is often preceded by a mucosal lesion called an oral potentially malignant disorder (OPMD). Many plant-derived compounds are of value in medicine. The objectives of this study were to develop a soluble mucoadhesive film containing α-mangostin (α-MG), a compound extracted from the peel of mangosteen fruit, and determine its activities against oral cancer cells, against human papillomavirus type 16 (HPV-16) pseudovirus, and its anti-inflammatory properties.

**Methods:**

A soluble mucoadhesive film containing α-MG was prepared. Oral squamous carcinoma cell line (SCC25), murine macrophage cells (RAW264.7), and human gingival fibroblast cell line were cultured. Anticancer activity and viability of SCC25 cells in response to α-MG film solution were determined by MTT assay. HPV-16 pseudovirus was constructed and effects of the film solution on attachment and post-attachment steps of the infection were investigated. Anti-inflammatory activity was assessed by nitric oxide (NO) inhibition. Fibroblast cell migration was determined by in vitro scratch assay.

**Results:**

The soluble α-MG film showed cytotoxic effects on SCC25 cells in concentration > 125 µg**/**ml with IC_50_ of 152.5 µg/ml. Antiviral activity against HPV-16 pseudovirus was observed at attachment step, but not at post-attachment step. The film also possessed a strong anti-inflammatory effect and promoted wound healing without cytotoxicity.

**Conclusions:**

Mucoadhesive film containing α-MG has a cytotoxic effect on oral squamous carcinoma cell line and an inhibitory effect on HPV-16 pseudovirus at attachment step. The α-MG film also shows a potent anti-inflammatory activity and enhances wound healing. Thus, the soluble α-MG film may have a potential role in treating oral cancer.

## Background

Despite advances in diagnosis and treatment, oral squamous cell carcinoma (OSCC) is still a significant oral health problem [1, 2]. It accounts for more than 90% of all oral cancers [3], with five-year survival rate of around 60% [4, 5]. OSCC is often preceded by a lesion called oral potentially malignant disorder (OPMD), which includes a variety of conditions associated with chronic irritation and inflammation such as leukoplakia/erythroplakia, and oral lichen planus.

Plants are excellent sources of new bioactive compounds. Mangosteen pericarp contains various phytochemicals, which are used in traditional medicines [6]. Xanthones are the phytochemical groups in the mangosteen pericarp that are associated with different biological activities including cardioprotective, antioxidant, anti-inflammatory, antibacterial, anti-allergy, and anticancer activities [7]. Of all xanthones derived from the mangosteen pericarp, α-MG is the most abundant and shows potent anticancer activities against many types of cancer cell lines including OSCC cell lines [8].

In addition to anticancer activity, other bioactivities of mangosteen peel extract have been reported. For instance, it has been demonstrated to reduce inflammation related to gingivitis in rats [9]. A study by Kresnoadi et al. [10] revealed that mangosteen pericarp extract could reduce the inflammation of post-tooth extraction in guinea pigs. Antimicrobial activity of α-MG against bacteria and fungi has been previously documented [11]. Moreover, antiviral activity of α-MG has also been reported [12, 13].

Currently, there is no reliable molecular hallmark that can predict malignant transformation of OPMD. Thus, the preventive therapy of malignant transformation of the lesions is a reasonable approach. Because α-MG has been shown to possess various bioactivities, we hypothesized that mucoadhesive film containing α-MG would provide anticancer, anti-HPV16 and anti-inflammatory activities and promote wound healing.

## Methods

### α-mangostin

α-MG used in this study was purchased from a local company in Thailand (Chemipan, Bangkok, Thailand). The compound was derived from pericarp of mangosteen extract (food grade).

### Preparation of mucoadhesive film containing α-mangostin

A soluble mucoadhesive film containing active ingredients α-MG (5 mg/ml) was prepared by modifying the method previously described [14]. The film contained α-MG 20%, hydroxypropyl methylcellulose 1.2% (HPMC E15, Methocel F4M, Dupont, Delaware, USA), polyethylene glycol 400 1.35% (Chemipan, Bangkok, Thailand), glycerin 1.6%, xylitol 1%, citric acid 0.04% and deionized water 74.8%. The HPMC was dissolved in water followed by polyethylene glycol, glycerin, xyletol and citric acid and α-MG. The mixture was cast on a glass plate 75 × 15 mm in size and dried in an oven at 70˚C for 24 h.

### Cell culture conditions

#### Cell culture

The murine macrophage cells (RAW264.7) were purchased from American Type Culture Collection (ATCC). RAW264.7 cell lines were grown in RPMI-1640 medium, supplemented with 10% fetal bovine serum (FBS), 0.1% sodium bicarbonate and 1% penicillin–streptomycin. A human embryonic kidney cell line, 293FT (Invitrogen, Carlsbad, CA, USA) was grown in Dulbecco’s modified Eagle’s medium (DMEM; Gibco-Life Technologies, Grand Island, NY, USA) with 10% FBS (Himedia, Mumbai, India), 0.1 mM MEM non-essential amino acids (Gibco), 6 mM L-glutamine (Gibco) and 500 µg/mL G418 sulfate (Calbiochem, Merck Biosciences Ltd., Nottingham, UK). The SCC25 cell line, Homo Sapiens tongue squamous cell carcinoma, purchased from ATCC, was maintained in culture system according to the previously described procedures [15], and cultured in DMEM (Gibco, NY, USA) with supplement of 15% FBS (Gibco) and 100 U/ml antibiotic–antimycotic (Gibco). All cell lines were maintained at 37 °C in a humidified atmosphere of 5% CO_2_.

### Cell viability assay

Cytotoxicity was determined by the MTT assay as previously described [16]. In brief, the RAW264.7, 293FT, and SCC25 cell lines were cultured before being harvested with 0.25% trypsin–EDTA and then diluted in a fresh medium. The cells were seeded in 96-well plates with 2 × 10^4^ (for 293FT cell line) and 1 × 10^4^ cells/well (for RAW264.7 and SCC25 cell lines) and allowed to adhere at 37 °C for 24 h. After that the medium was replenished with fresh medium (RPMI-1640 for RAW264.7, DMEM for 293FT and SCC25) along with the dissolved α-MG mucoadhesive film solution (6.25–50 μg/ml for RAW264.7 cells, 0.044–4400 μg/ml for 293FT cells and 1.95–259 μg/ml for SCC25 cells) and was then incubated for 24 h. Ten microliters, of MTT solution (5 mg/ml in PBS) was added to the 96-well plates. After 2 h of incubation, the medium was removed, and DMSO (200 μl) was added to each well to dissolve the formazan solution. It was then measured with a microplate reader at 540–570 nm (Multiskan™ FC; Thermo Fisher Scientific, Waltham, MA, USA). The test samples were considered cytotoxic when the optical density (OD) of the sample-treated group was less than 80% of that in the control group. Cell viability was calculated using the following equation:

$$\% {\text{Cell viability}} = [{\text{OD}}_{{{\text{sample}}}} /{\text{OD}}_{{{\text{control}}}} ] \times 100$$

### Antiviral activity against HPV-16 pseudovirus

#### HPV-16 pseudovirus production

The 293FT cells were seeded in 25 cm^2^ culture flask at 3 × 10^5^ cells/flask and maintained for 4 days. The cells were co-transfected with p16SheLL (6 μg) and pfwB (6 μg) plasmids which were kindly provided by John T. Schiller (Laboratory of Cellular Oncology, Bethesda, MD, USA) using Lipofectamine 20,000 (Invitrogen, Carlsbad, CA, USA) for 6 h. After 4 days post-transfection, the transfected cells were harvested and lysed in a lysis buffer containing 0.5% Brij 58 (Sigma-Aldrich, St. Louis, MO, USA), 0.2% RNase A (bovine pancreas, Sigma Chemical Company, St. Louis, MO, USA), 9.5 mM MgCl_2_ in PBS. The lysed cells were incubated at 37 °C for 24 h and then chilled on ice for 5 min and kept at -80 °C for a long-term storage until use.

#### Determination of HPV-16 pseudovirus titer

The 293FT cells were seeded in a 96-well plate at 3 × 10^3^ cells/well and incubated for 6 h. HPV-16 pseudovirus stock was diluted to 1:5000, 1:10,000, 1:20,000 and 1:40,000. Each diluted viral stock was added into the cells and incubated at 37 °C, 5% CO_2_ for 4 days. The 293FT cells which were infected by HPV-16 pseudovirus, displayed green fluorescence under a fluorescent microscope (Olympus BX51, Olympus Co., Ltd., Tokyo, Japan). The cells were harvested and then counted by a hemocytometer (Marienfeld GmbH, Marienfeld, Germany) under light and fluorescent microscope (Olympus). The infectious titer is interpreted as transducing units (TU)/ml and was calculated by the formula:

$$\begin{aligned} {\text{Viral titer}}({\text{TU}}/{\text{ml}}) &= \% {\text{infection}} \times ({\text{cell density seeded}}) \\ &\quad\times {\text{dilution factor}}/{\text{volume of viral stock}} \end{aligned}$$

#### Cytotoxicity

The 293FT cells were seeded into a 96-well plate at 2 × 10^4^ cells/well and then incubated for 24 h. Prior to cell viability test, the α-MG mucoadhesive film was dissolved in the culture medium resulting as a solution sample. The cells were then treated with various concentrations of the film solution sample (0, 0.4, 0.6, 0.8, 1.0, 2.0 and 4.0 μg/ml) for 48 h.Ten microliters of 5 mg/ml MTT (Sigma, St. Louis, MO, USA) was added to each well. After 4 h, the medium was removed and the water-insoluble purple formazan particles were dissolved in 100 μl DMSO solution. The absorbance was read at 540 nm with a microplate reader (Multiskan GO, ThermoScientific, USA).

#### Determination of anti-HPV-16 pseudovirus infection at attachment step

The 293FT cells were seeded in a 96-well plate at 3 × 10^3^ cells/well and cultured for 4 days. HPV-16 pseudoviruses (MOI 0.05 and 0.5) were treated with or without the α-MG mucoadhesive film solution (0, 0.2, 0.4, 0.8, 2.0, 4.0 and 8.0 μg/ml) at 37 °C for 1 h. The treated pseudoviruses were adsorbed on the cells and incubated at 37 °C for 4 h. Unattached pseudoviruses were removed and then incubated in complete medium at 37 °C for 72 h.

#### Determination of anti-HPV-16 pseudovirus infection at post-attachment step

The 293FT cells were seeded in a 96-well plate at 3 × 10^3^ cells/well and cultured for 4 days. HPV-16 pseudoviruses (MOI 0.05 and 0.5) were adsorbed on the cells and incubated at 25 °C for 2 h to allow HPV-16 pseudovirus to bind to their receptors but not enter into the cells. After removing unattached pseudoviruses, the cells were maintained in complete media with or without the α-MG mucoadhesive film solution (0, 0.2, 0.4, 0.8, 2.0, 4.0 and 8.0 μg/ml) at 37 °C for 72 h.

To determine anti-HPV-16 pseudovirus infection both at attachment step and at post-attachment step, heparin (400 µM) was used as a positive control. HPV-16 pseudovirus-infected cells were detected by observing green fluorescence under a fluorescent microscope. The cells were harvested and then counted by a hemocytometer under light and a fluorescent microscope to analyze the percentage of inhibition.

### In vitro anti-inflammatory study

#### Nitric oxide (NO) inhibition

Anti-inflammatory activity of the α-MG mucoadhesive film solution was measured by a method modified from Sae-Wong et al. [16]. In brief, the RAW264.7 macrophage cell lines were seeded in 96-well plates (1 × 10^4^ cells/well) and allowed to adhere for 2 h at 37 °C in a humidified atmosphere containing 5% CO_2_. Two hours later, the non-adherent cells and medium were removed and the adherent cells were cultured in a fresh medium containing 1 μg/ml LPS (lipopolysaccharides L4005, Sigma-Aldrich, Missouri, USA) and various concentrations of the α-MG mucoadhesive film solution for 24 h. NO production in each well was assessed by measuring the accumulation of nitrite (NO_2_) in the culture medium using Griess reagent. One hundred μl of supernatant was mixed with 100 μl of Griess reagent and the optical density (OD) was detected at 570 nm. L-nitro-arginine (L-NA), which is NO synthase inhibitor, was used as a positive control (6.25–50 μg/ml). The percentage of inhibition of NO production was calculated using the following equation.

$${\text{NO Inhibition(}}\% {)} = \frac{{\left[ {\left( {{\text{control}} - {\text{blank of control}}} \right) - \left( {{\text{sample}} - {\text{blank of sample}}} \right)} \right]}}{{\left( {{\text{control}} - {\text{blank of control}}} \right)}}$$

### In vitro scratch assay

Human gingival fibroblast cell line was seeded into 6-well plates at a density of 1 × 10^6^ cells/well. A linear scratch was generated with a sterile pipette tip in the monolayer when it was confluently formed. Cellular debris was removed by washing three times with 3 ml PBS and replaced with 2 ml of complete medium containing the α-MG mucoadhesive film solution (29.20 mg/ml), while complete medium without the film solution served as a negative control. Photographs were taken at a 10 × magnification using a microphotograph on day 0, then plates were incubated at 37 °C with 5% CO_2_ and photographs were taken at days 1 and 2. The images acquired for each sample were further analyzed quantitatively by using computing software ImageJ [17]. The distance of each scratch closure was determined by comparing the images from day 0–2, and the percentage migration rate was calculated. Two scratches were made in each well (left and right) and six random microscopic fields were considered per scratch. The average of the left scratch and the right scratch were taken separately. The percentage of migration was calculated for the left scratch and then the right scratch using the following equation:

$$\% {\text{Migration rate}} = \frac{{{\text{average distance between scratch day 0}} - {\text{average distance between scratch day 1}}}}{{\text{average distance between scratch day 0}}}$$

### Statistical analysis

Statistical analysis was performed using Prism5 software (GraphPad, San Diego, CA, USA). Comparisons between untreated and treated groups were investigated by Student’s t-tests. The data are expressed as mean ± SEM (standard error of the mean). The symbols *, ** and *** are denoted as statistically significant differences (*P* < 0.05, 0.01 and 0.001, respectively).

## Results

### Cytotoxicity of mucoadhesive film containing α-mangostin

**Fig. 1:**
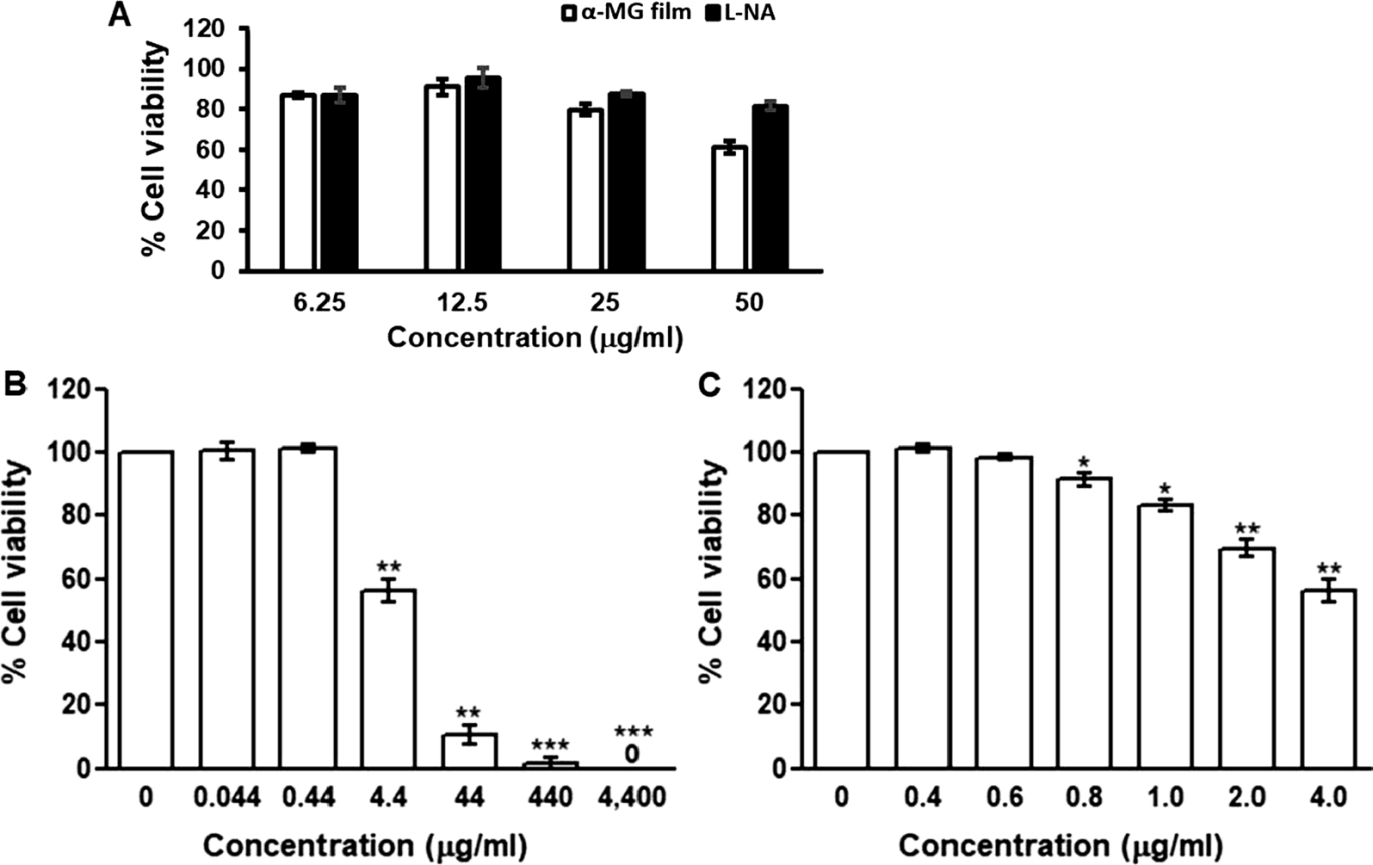
Cytotoxicity of mucoadhesive film solution (6.25–50 μg/ml) containing α-mangostin (α-MG, 5 mg/ml) by MTT assay on RAW264.7 macrophage cell line (**A**), on 293FT cells at ten-fold dilution 0 to 4400 µg/ml (**B**) and at 0–4.0 µg/ml (**C**). Data presented as mean ± SEM (*n* = 4). Differences in percentage of cell viability were statistically analyzed using One-way ANOVA. *, ** and *** denote statistically significant differences as *P* < 0.05, 0.01 and 0.001, respectively

The cytotoxicity of the α-MG mucoadhesive film was determined by MTT assay using murine macrophage cell line (RAW264.7 cells). The IC_50_ of the α-MG film and L-NA was 64.51 μg/ml and 125.80 μg/ml, respectively. The film solution was not toxic to the cells, with 80% survival at the maximum concentration of 25 µg/ml concentrations (Fig. 1A). An increase in concentration of the film solution led to a decrease in cell viability, and the toxicity was observed at the concentration of ≥ 50 µg/ml. In contrast, L-NA standard was applied to the cells at a concentration of 50 µg/ml and the cell viability remained more than 80%.

The cytotoxic effect of the α-MG mucoadhesive film was also determined in 293FT cells. Various concentrations of the film solution (0–4400 µg/ml) were screened and analyzed for 48 h after treatment. The concentration of the film at ≤ 4.4 μg/ml had ≥ 50% of cell viability (Fig. 1B). Subsequently, the treatment with the concentration of the film at 0, 0.4, 0.6, 0.8, 1.0, 2.0 and 4.0 μg/ml was performed. The cell viability was not decreased with the concentration of the film solution at ≤ 0.8 µg/ml (Fig. 1C).

### Anticancer activity of mucoadhesive film containing α-mangostin

**Fig. 2:**
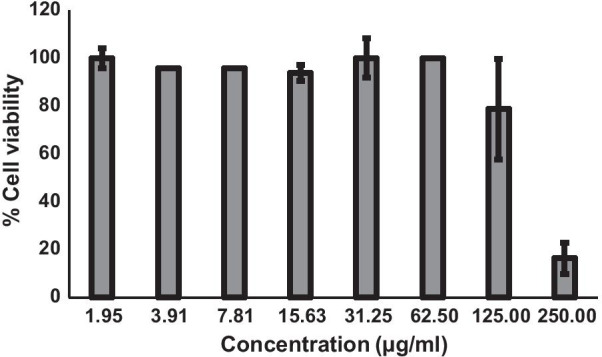
Effects of mucoadhesive film containing α-MG on viability of SCC25 cell line. Viability of SCC25 cell line in response to the α-MG film solution was determined by MTT assay. Cytotoxic effects was observed when the concentration of the film was > 125 µg**/**ml with the IC_50_ of 152.5 µg/ml

Viability of SCC25 cell line in response to the mucoadhesive film containing α-MG was determined by MTT assay. The results showed cytotoxic effects on the cell viability when the concentration of the film solution was > 125 µg**/**ml with the IC_50_ of 152.5 µg/ml (Fig. 2).

### Antiviral activity of mucoadhesive film containing α-mangostin

#### Effect on HPV-16 pseudovirus infection at attachment step

**Fig. 3:**
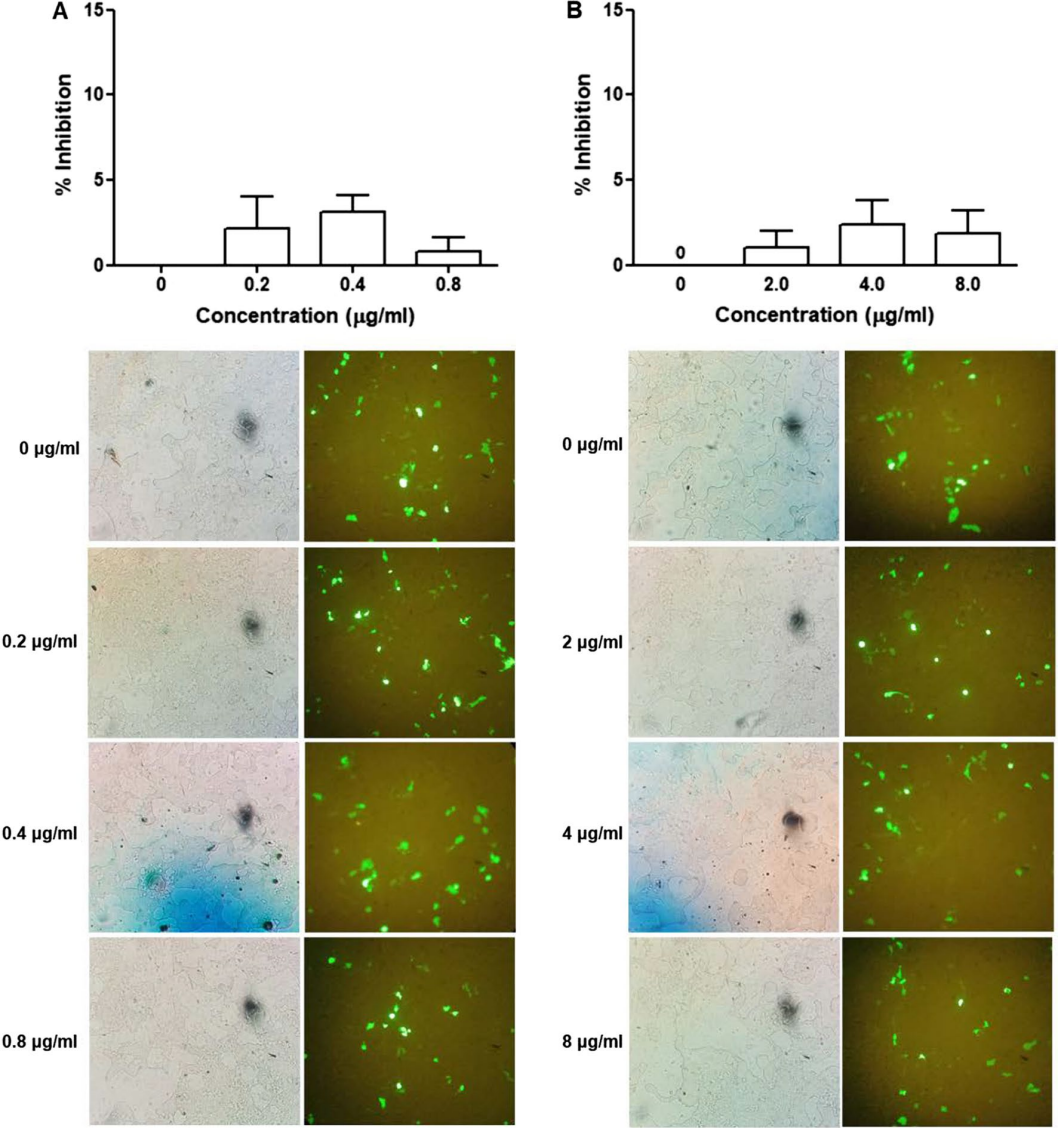
Effect of mucoadhesive film containing α-MG on HPV16 pseudovirus at attachment step. The 293FT cells were cultured in a 96-well plate for 6 h and then treated with various concentrations of the α-MG film solution at 0, 0.2, 0.4 and 0.8 μg/ml (non-cytotoxic conditions) (**A**) and 0, 2.0, 4.0 and 8.0 μg/ml (cytotoxic concentrations) mixed with HPV16 pseudovirus (MOI 0.05) (**B**). Percentage of inhibition was calculated from the number of HPV16 pseudovirus-infected cells relative to all cells counted by hemocytometer and compared to untreated cells

To study the effects of the mucoadhesive film containing α-MG on HPV-16 pseudovirus at attachment step, HPV-16 pseudovirus at MOI 0.05 and 0.5 was treated with or without the film at concentrations of 0, 0.2, 0.4, 0.8 μg/ml (non-cytotoxic concentrations), and 0, 2.0, 4.0 and 8.0 μg/ml (cytotoxic concentrations), incubated for 1 h and added to the cells. After 72 h treatment, the result showed that the α-MG film reduced the percentage of HPV-16 pseudovirus-infected cells in the attachment step only in MOI 0.05 (Fig. 3) but not in MOI 0.5 (data not shown). However, no significant difference was found in contrast to untreated cells.

#### Effect on HPV-16 pseudovirus infection at post-attachment step

**Fig. 4:**
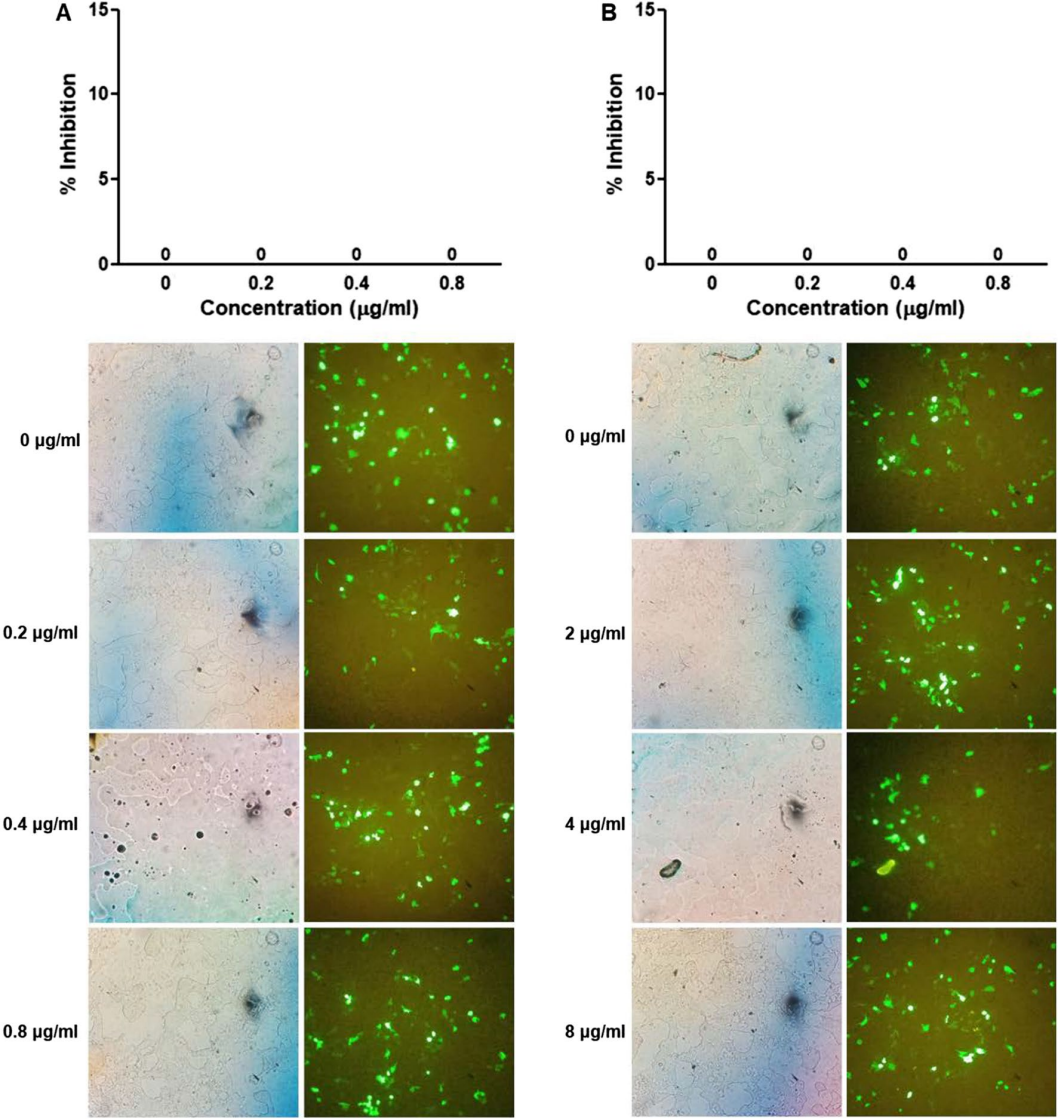
Effect of mucoadhesive film containing α-MG on HPV16 pseudovirus at post-attachment step. The 293FT cells were cultured in 96-well plate for 6 h and then were added with HPV16 pseudovirus (MOI 0.05) for 4 h before treated with various concentrations of the α-MG film solution at 0, 0.2, 0.4 and 0.8 μg/ml (non-cytotoxic condition) (**A**) and 0, 2.0, 4.0 and 8.0 μg/ml (cytotoxic concentration) (**B**). Percentage of inhibition was calculated from the number of HPV16 pseudovirus-infected cells relative to all cells counted by hemocytometer and compared to untreated cells

To study the effects of the mucoadhesive film containing α-MG on HPV-16 pseudovirus at post-attachment step, HPV-16 pseudoviruses at MOI 0.05 and 0.5 were adsorbed on 293FT cells and incubated for 4 h to allow pseudoviruses to bind to their receptors on the cell surface. The α-MG films solution at concentrations of 0, 0.2, 0.4, 0.8 μg/ml (non-cytotoxic concentrations), and 0, 2.0, 4.0 and 8.0 μg/ml (cytotoxic concentrations) were added to pseudovirus-attached cells and incubated for 72 h. The result showed that the percentage of HPV-16 pseudovirus-infected cells in the post-attachment step was not decreased in either MOI 0.05 (Fig. 4) or 0.5 infection (data not shown).

### Anti-inflammatory activity of mucoadhesive film containing α-mangostin

**Fig. 5:**
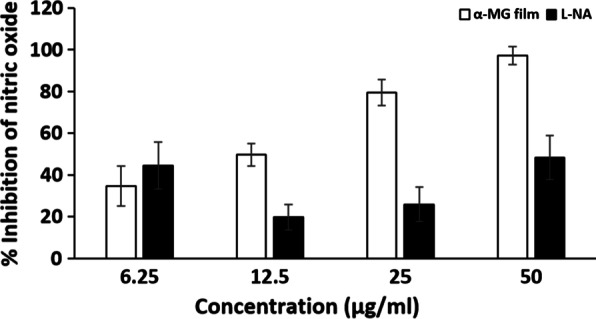
Anti-inflammatory activity of mucoadhesive film containing α-MG. Anti-inflammatory activity of mucoadhesive film containing α-MG (5 mg/ml) was determined by percent inhibition of NO in RAW264.7 macrophage cell lines. The percentage of inhibition of NO production in the cells after treated with the α-MG film solution (6.25–50 μg/ml) was dose-dependent. L-NA served as positive control. Data presented as mean ± SEM (*n* = 4)

In order to determine anti-inflammatory activity, percent inhibition of NO was assessed. The anti-inflammatory activity of the mucoadhesive film containing α-MG was evaluated in RAW 264.7 cells. The film demonstrated the inhibition of NO in a dose-dependent manner (Fig. 5). Of interest, NO inhibition activity of the α-MG film was found to be better than L-NA standard. The IC_50_ of the α-MG film was about four times less concentrated than that of L-NA standard.

### Effects of mucoadhesive film containing α-mangostin on cell migration

**Fig. 6:**
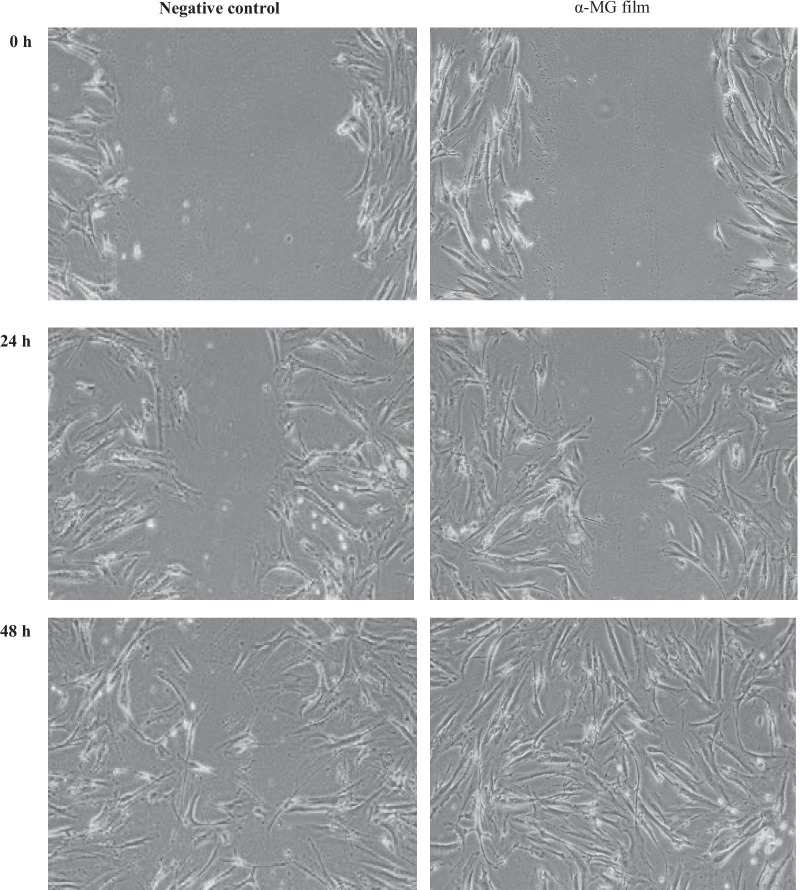
Measurement of cell migration in the in vitro scratch assay. A human gingival fibroblast layer subjected to scratch and treated with mucoadhesive film containing α-MG (29.20 μg/ml) at 0, 24 and 48 h after incubation. The cell migration was more efficient in the presence of the α-MG film solution than the control at both 24 h and 48 h

Measurement of cell migration was determined in vitro by scratch assay. A human gingival fibroblast layer was scratched and treated with the α-MG mucoadhesive film solution (29.20 µg/ml) at 0, 24 and 48 h after incubation. It was noted that cell migration was more efficient in the presence of the film than the control both at 24 h and 48 h (Fig. 6).

## Discussion

This study demonstrated that mucoadhesive film containing α-MG affects viability of oral cancer cell line and seems to inhibit HPV-16 pseudovirus at the attachment step of the infection, but not at the post-attachment step. The α-MG film also shows strong anti-inflammatory activity and may promote wound healing without cytotoxic effects at a therapeutic dose.

A previous study by Kwak et al. [8] reported that α-MG inhibits cell proliferation and induces cell death in OSCC cells in a dose- and time-dependent manner with little to no effect on normal human periodontal ligament cells. Moreover, α-MG was shown to decrease cell viability by inducing apoptosis and cell cycle arrest in YD-15 tongue mucoepidermoid carcinoma cells [18]. Cytotoxic effect of α-MG was also reported in other types of cancer cells, including colon and prostate cancers [19, 20]. In our study, the mucoadhesive film containing α-MG shows anticancer activity at a relative high dose when compared to that tested with the compound alone reported in the literature [8]. This may be due to the fact that some of the α-MG may be entrapped by other ingredients when the film is formulated, and thus its anticancer activity might be reduced. Optimization of the mucoadhesive film will be needed for enhancing the cytotoxic effect observed in this study [21]. The difference may also result from the difference in cell lines used in the study.

In the present study, the mucoadhesive film containing α-MG seems to inhibit HPV-16 pseudovirus at the attachment step of infection. Previous studies reported that the frequency of HPV virus in carcinoma and potentially malignant cases ranges from 0 to 100% [22]. HPV has been associated with head and neck squamous cell carcinoma. However, some studies revealed that only a small proportion of OSCC appears to be caused by HPV [23, 24]. The prevalence of high risk HPV in OSCC cases from various countries has been reported, with Asian countries tending to have a lower prevalence of high risk HPV compared to those reported from western regions [25, 26]. The difference in frequencies in the studies has been attributed to the type of samples collected, methodology used to study the samples, and the selected patient group [27, 28]. Thus, the role of HPV infection on promoting malignant transformation of some OPMD lesions into OSCC remains unclear and should be further investigated [27].

In the present study, the mucoadhesive film containing α-MG shows more potent anti-inflammatory activity than that of L-NA standard, and seems to promote wound healing. These effects may help to control malignant transformation as OPMD include a variety of lesions that are commonly associated with chronic irritation and inflammation such as leukoplakia/ erythroplakia, or ulcerative lesions in some cases of oral lichen planus and discoid lupus erythematosus. As chronic inflammation is a well-known risk factor for malignant changes [29], transformation of lesions related to inflammatory disorders such as oral lichen planus and discoid lupus erythematosus into OSCC may be preventable to some extent by reducing inflammation. In particular, in those with risk factors, which may synergistically contribute to OSCC, such as smoking, alcohol consumption, use of smokeless tobacco and betel quid chewing [30], the α-MG film may be applied to control inflammation and promote healing of those OPMD lesions and thus may help to prevent progression of the lesions into OSCC.

## Conclusions

This study demonstrated that the mucoadhesive film containing α-MG has a strong anti-inflammatory effect and promotes wound healing without cytotoxicity at a therapeutic dose. The film also shows a cytotoxic effect on the viability of SCC25 cell lines and inhibits HPV-16 pseudovirus infection at the attachment step. Thus, the mucoadhesive film containing a-MG may have a potential role in oral cancer treatment. Further studies should be performed in other SCC cell lines and other HPV genotypes associated with OSCC.

## Data Availability

The datasets generated and/or analyzed during the current study are not publicly available, but are available from the corresponding author on reasonable request.

## Abbreviations

α-MG: α-Mangostin
ATCC: American type culture collection
DMEM: Dulbecco’s modified Eagle’s medium
DMSO: Dimethyl sulfoxide
EDTA: Ethylenediaminetetraacetic acid
FBS: Fetal bovine serum
HPMC: Hydroxypropyl methylcellulose
HPV-16: Human papilloma virus type 16
MTT: (3-(4,5-Dimethylthiazol-2-yl)-2,5-Diphenyltetrazolium Bromide)
NO: Nitric oxide
OD: Optical density
OSCC: Oral squamous cell carcinoma
PBS: Phosphate buffer saline
OPMD: Oral potentially malignant disorder
TU: Transducing units
